# Abnormalities in collagen composition may contribute to the pathogenesis of hemorrhoids: morphometric analysis

**DOI:** 10.1007/s10151-014-1238-5

**Published:** 2014-11-09

**Authors:** Y. Y. Nasseri, E. Krott, K. M. Van Groningen, M. Berho, M. C. Osborne, S. Wollman, E. G. Weiss, S. D. Wexner

**Affiliations:** 1 Department of Colorectal Surgery, Cleveland Clinic Florida, 2950 Cleveland Clinic Blvd., Weston, FL 33331 USA; 2 Klinik für Allgemein-, Visceral-und Transplantationschirurgie, Universitätsklinikum Aachen, Aachen, Germany

**Keywords:** Hemorrhoids, Collagen, Pathogenesis, Connective

## Abstract

**Purpose:**

While hemorrhoidal disease is common, its etiology remains unclear. It has been postulated that disturbances in collagen lead to reduced connective tissue stability, and in turn to the development of hemorrhoidal disease. We aimed to compare the quality and quantity of collagen in patients with hemorrhoidal disease versus normal controls.

**Methods:**

Specimens from 57 patients with grade III or IV internal hemorrhoids undergoing hemorrhoidectomy between 2006 and 2011 were evaluated. Samples from 20 human cadavers without hemorrhoidal disease served as controls. Quality of collagen was analyzed by collagen I/III ratio, and quantity of collagen was determined by collagen/protein ratio. The study group was subdivided into gender and age subgroups.

**Results:**

The male:female ratios in the study and control groups were 30:27 and 10:10, respectively. Median age was significantly less in the study group [46.9 years (range 20–69)] compared to the control group [76 years (range 46–90)] with *P* < 0.05. Tissues from patients in the study group had significantly lower collagen I/III ratio as compared to the control group (4.4 ± 1.1 vs. 5.5 ± 0.6; *P* < 0.0001). Nevertheless, despite a trend toward lower collagen/protein ratio in the study group, it did not reach statistical significance (57 ± 42.4 vs. 73 ± 32.5 g/mg; *P* = 0.167). There was no difference in collagen I/III or collagen/protein ratios among different age groups and genders.

**Conclusions:**

Hemorrhoidal tissues from patients with hemorrhoidal disease appear to have reduced mechanical stability as compared to normal controls.

## Introduction

Hemorrhoids have been described as far back as the pre-Christian era [1]. In 1830, de Montégre [2] assembled an early literature review of 78 articles on hemorrhoids published between 1582 and 1817. His manuscript remarked how little was known about the overall prevalence and risk factors of hemorrhoidal disease; not much changed during the past 200 years. Although most colorectal surgeons recognize that hemorrhoids are common, they are unaware of their true prevalence. Previous studies have reported rates ranging from 4.4 [3] to 86 % in the general population [4].

Hemorrhoidal disease is defined as the symptomatic enlargement and distal displacement of the normal anal cushions [5]. The main theory regarding the pathophysiology of hemorrhoidal disease suggest that they are the result of abnormal dilation of veins of the internal hemorrhoidal venous plexus, abnormal distention of the arteriovenous anastomosis, and prolapse of the cushions and the surrounding connective tissue. Numerous factors have been linked with hemorrhoidal disease including inadequate fiber intake, prolonged lavatory sitting, constipation, diarrhea, and pregnancy. Family history of hemorrhoidal disease has also been suggested as a possible etiology. However, there is no conclusive evidence of hereditary predisposition.

Thompson [6] and Aigner et al’s [7] studies have shown that hemorrhoidal disease is the consequence of disintegration of muscular and elastic components, leading to distal shift of the vascular padding, and increased arterial blood flow of the terminal branches of the superior rectal artery, respectively. Further, it has been suggested that the degradation of the extracellular matrices in hemorrhoids during aging is a decisive pathway in the development of hemorrhoidal disease [8]. The elastic and tensile strength of hemorrhoids, as provided by elastic fibers and collagen, respectively, and collagen metabolism, have also been postulated to have effects on the development of hemorrhoidal disease [9]. Willis et al. [9] analyzed the quantity and quality of collagen formation in the corpus cavernosum recti in patients with grades III/IV internal hemorrhoids in comparison with a control group of individuals without hemorrhoidal disease. They found that disturbances in collagen I/III and collagen/protein ratios lead to reduced connective tissue stability, possibly contributing to the development of hemorrhoidal disease. Nevertheless, their study analyzed a relatively small number of patients with hemorrhoidal disease.

We sought to study a larger cohort of patients and compare the quality and quantity of collagen in patients with internal hemorrhoidal disease (study group) versus normal controls (control group) and among different genders and age groups within the study group.

## Methods

### Patients

Patients with grade III or IV internal hemorrhoids who underwent standard hemorrhoidectomies between 2006 and 2011 were identified. Specimens from these patients were collected and fixed in 10 % formalin and immediately embedded in paraffin for later analysis. The study group was further subdivided into one of two genders and one of three age groups (20, 40, and 60 s).

The control group was made up of 20 human cadavers without hemorrhoidal disease. In review of the past medical histories of these cadavers, hemorrhoidal disease was never listed as a condition they had. All 20 cadavers died of natural causes and were made available from the Institute of Anatomy of the RWTH Aachen, Germany. The cadavers were analyzed immediately after their arrival at the institute, and the anal and deep rectal skin and submucosa were resected by traditional hemorrhoidectomy. Specimens from the 20 controls were collected and fixed in 10 % formalin after the procedure and immediately embedded in paraffin for later analysis. An Institutional Review Board at the Cleveland Clinic Florida approved this HIPAA compliant study. No identifiers were included in our study.

### Collagen/protein ratio

The relative amount or quantity of collagen was measured by the collagen/protein ratio. Fifteen-micrometer-thick specimens sealed in paraffin were collected from each group and placed in test tubes. After the paraffin was removed, the slices were stained with Sirius red and Fast green dyes (Polysciences, Warrington, PA, USA). The samples were then rinsed several times with distilled water until the supernatant was colorless. Next, the dyes were eluded from the sections by using 0.1 N NaOH in absolute methanol. The resulting fluid was then immediately measured in a spectrophotometer at the wavelengths corresponding to the maximum absorbance of Sirius red (535 nm) and Fast green (605 nm). Results were expressed as the ratio of collagen (g) to non-collagenous protein (mg).

### Collagen type I/III ratio

The quality of the collagen from the specimens was evaluated by the collagen I/III ratio by means of cross-polarization microscopy at the same institution. Each specimen was sliced into five-micrometer sections that were then stained for 1 h in Picrosirius solution (0.1 % solution of Sirius Red F3BA in saturated aqueous picric acid, pH 2). The sections were then washed for 2 min in 0.01 N HCl, dehydrated, cleared, and mounted in synthetic resin. Thicker collagen I fibers were stained in re-orange shades while thinner type III appeared as pale-green shades. For every sample, 10 regions within the interface (400x, area 50 × 50 m) were captured by a digital camera (Olympus C-3030, Hamburg Germany). The varying amounts of collagen I/III ratios were obtained by analysis of the amount of collagen type I and III using digital image analysis software (Image-Pro Plus, Media Cybernetics, Silver Spring, Maryland, USA). Results are expressed as ratio of area of collagen type I and III.

### Statistics

Statistical analysis was performed by PGS Medical Statistics using SAS Statistical software, V9.3. For the descriptive analysis, the mean, standard deviation (SD), median, minimum, and maximum values (range), and the number of valid observations were calculated in each group. A student’s *t* test was used to compare the study and control groups and genders. Analysis of variance (ANOVA) was used to compare the three age groups for the collagen I/III ratio, while a Kruskal–Wallis test was used to compare the age groups for the collagen/protein ratio. A *P* value of <0.05 was used to indicate statistical significance.

## Results

The mean age of the study group was 46.9 (20–69), which was significantly lower than the mean age of the control group 76 (46–90) years with *P* < 0.05. The male:female ratios for the study and control groups were 30:27 and 10:10, respectively.

### Collagen type I/III ratio

**Fig. 1:**
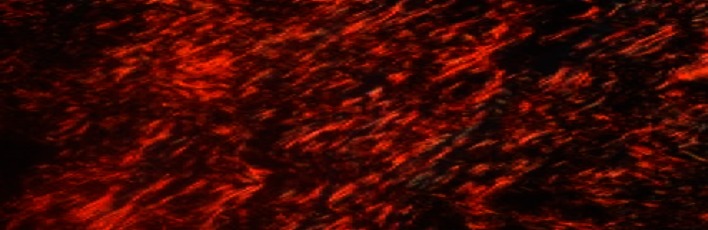
Sirius red—staining of collagen I (*red*) and collagen III (*green*) in a healthy control person

**Fig. 2:**
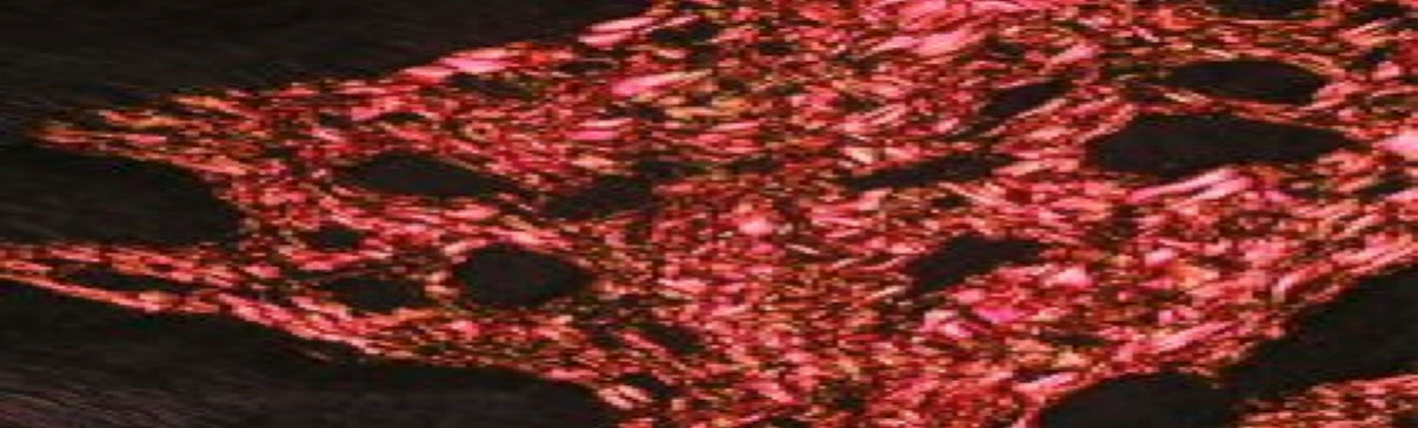
Sirius red—staining of collagen I (*red*) and collagen III (*green*) in a patient with hemorrhoidal disease

The mean collagen I/III ratio was significantly lower in patients with hemorrhoidal disease as compared to the control group (4.4 ± 1.1 vs. 5.5 ± 0.6; *P* < 0.001; Table 1). See Figs. 1 and 2 for representative comparative collagen I/III ratio slides. Within the study group, there was no significant difference in the collagen I/III ratio between men and women (4.4 ± 1.2 vs. 4.3 ± 1.0; *P* = 0.612) and among the 20 s (*n* = 17), 40 s (*n* = 18), and 60 s (*n* = 22) aged subgroups (4.3 ± 1.2, 4.8 ± 1.2, 4.4 ± 1.0; *P* = 0.124; Table 2).

**Table 1:** A direct comparison of study versus control groups

	M:F ratio	Mean age in years (range)	Collagen I/III ratio	*P* value (collagen I/III)	Collagen/protein ratio (g/mg)	*P* value (collagen/protein)
Study (*n* = 57)	30:27	46.9 (20–69)	4.4 ± 1.1	*P* < 0.001	57 ± 42.4	0.167
Control (*n* = 20)	10:10	76 (46–9)	5.5 ± 0.6		73 ± 32.5

**Table 2:** Comparison of different gender and age groups within the study group

Study (*n* = 57)	Collagen I/III ratio	*P* value (collagen I/III)	Collagen/protein ratio (g/mg)	*P* value (collagen/protein)
Gender				
Male (*n* = 30)	4.4 ± 1.2	0.612	50.8 ± 32.6	0.258
Female (*n* = 27)	4.3 ± 1.0		64 ± 50.9	
Age				
20 s (*n* = 17)	4.3 ± 1.2	0.124	65 ± 62.8	0.314
40 s (*n* = 18)	4.8 ± 1.2		46.3 ± 23.6	
60 s (*n* = 22)	4.1 ± 1.0		59.7 ± 34.4	

### Collagen/protein ratio

The mean collagen/protein ratio was not significantly lower in patients with hemorrhoidal disease as compared to the control group (57 ± 42.4 vs. 73 ± 32.5 g/mg; *P* = 0.167; Table 1). Within the study group, there was no significant difference in the mean collagen/protein ratio between men and women (50.8 ± 32.6 vs. 64 ± 50.9 g/mg; *P* = 0.258) and among the 20, 40, and 60 s aged subgroups (65 ± 62.8, 46.3 ± 23.6, 59.7 ± 34.4 g/mg; *P* = 0.124; Table 2).

## Discussion

Collagen is the major insoluble fibrous protein in the extracellular matrix and connective tissue and is the single most abundant protein in the animal kingdom. While there are at least 16 types of collagen, 80–90 % of the collagen in the body consists of types I, II, and III [10]. Type I collagen fibers have immense tensile strength and can withstand enormous forces, while type III collagen are thinner and more immature [10, 11]. The strength and quality of connective tissue is primarily determined by the amount and ratio of collagens Type I and III. Decreased Type I to III collagen ratio translates into decreased amount of cross-linking and hence, reduced mechanical stability of connective tissue.

Our data clearly indicated a decrease in Type I/III collagen ratio in patients with hemorrhoidal disease as compared to normal controls. This finding may link reduced mechanical stability and tensile strength in extracellular matrix with development of hemorrhoidal disease. Our study further demonstrated a lower trend in the collagen/protein ratio in patients with hemorrhoidal disease, although this did not reach statistical difference as was seen in the Willis et al. [9] study. This difference can be attributed to difference in the power of the two studies.

Although hemorrhoidal disease has been associated with older age, we failed to demonstrate any difference in quality or quantity of hemorrhoids among different age groups within the study group. This finding may be due to either the small number of patients in each age group or to the fact that only patients with known hemorrhoidal disease were compared. Perhaps there is a true degradation in collagen with age in the general population, but due to genetic/hereditary factors, patients with hemorrhoidal disease are subject to earlier and more rapid tissue degradation.

The previously held belief that connective tissue disease directly correlates with hernias and genitourinary prolapse may also hold true with hemorrhoidal disease [12, 13]. As mentioned in the study by Willis et al., we too believe that hemorrhoidal turnover and degradation is likely the sequelae of genetic, metabolic, and environmental components.

One of the limitations of our study is the relatively small number of control patients. One must bear in mind that it is difficult to find hemorrhoidal tissue from live surgical patients without hemorrhoidal disease as those are usually found in patients who have undergone abdominoperineal resections. Tissues from these patients would not be ideal for the control group as they are usually from patients with inflammatory bowel disease or low rectal or anal cancers, following immunosuppressive use and chemoradiation, respectively; as such, we had to limit our control group to cadaver tissues of patients who died of natural causes. A possible criticism may be that cadaver tissues are different and not completely comparable with live human tissue due to possible differences in processing and preservation, as well as natural decay of tissues following death. Moreover, the German cadavers’ collagen composition may differ from the control group in Florida due to genetic influences and patient location. There is also a large difference in age between the study and control groups. While the use of cadaver tissue as our controls may be a potential weakness, it could also add strength to our study for various reasons. First, we were able to show that the control group had better quality collagen despite being at an advanced age with postmortem processing of tissue and subsequent morphological changes. Secondly, one could hypothesize that age and postmortem processing may lead to lower quality collagen, but we found this correlation not to be true. These findings support our point that genetic and hereditary factors may lead to reduced quality collagen and thus an earlier onset of hemorrhoidal disease. Within this study, we did not focus on collagen-vascular disorders such as Ehlers–Danlos or osteogenesis imperfecta and the belief that these disorders have a higher propensity to develop hemorrhoidal disease remains a subject of future investigation.

The main strength of our study lies in the relatively large number of patients with hemorrhoidal disease that were included. Additionally, our study likely gains further validity as it re-enforces the results of the study by Willis et al. relative to collagen I/III ratio and in the lack of difference among different age groups and genders.

The clinical relevance of our study may lie in the possible treatment options targeting enhancement and stimulation of collagen as extrapolated from other specialties. Bovine collagen injections have been used in the USA since the 1980s and have proven effective for correcting soft tissue contour irregularities [14]. Intra-anal collagen injections have shown to be simple, safe, and effective in the long-term treatment of fecal incontinence [15]. Additionally, fractional ablative lasers have been shown to induce wound healing and collagen remodeling responses [16]. Thus, collagen injections and lasers that enhance and stimulate collagen may prove effective in the treatment of hemorrhoidal disease.
